# Reply: PTHrP on MCF-7 breast cancer cells: a growth factor or an antimitogenic peptide?

**DOI:** 10.1038/sj.bjc.6601690

**Published:** 2004-03-02

**Authors:** R P Hoey, C Sanderson, J Iddon, G Brady, N J Bundred, N G Anderson

**Affiliations:** 1 University of Manchester, G186 Stopford Building, Oxford Road, Manchester M13 9PT, UK

**Sir**,

We appreciate the detailed critical analysis of our work by Maioli and Fortino, who have also been investigating the actions of PTHRP in MCF7 cells. The authors make a number of observations and criticisms of our work, to which we are glad to have the opportunity to respond.

The first point raised relates to our discussion of some work published by Falzon and Du (2000) regarding intracrine actions of PTHRP. It is true to say we considered that, because their data showing the intracrine effect required overexpression of PTHRP, such a mechanism was unlikely to operate in our system, in which we showed that endogenous PTHRP production was extremely low. We did not, however, question the physiological relevance of the Falzon group's findings and indeed suggested that it would be interesting to test for the presence of intracrine and autocrine activity in cells overexpressing both ligand and receptor.

The second point relates to the dose-responsiveness of MCF-7 cells to PTHRP. Maioli and Fortino's results show that, at PTHRP concentrations approximately five-fold higher than the highest dose employed in our study, PTHRP exerts an inhibitory effect on proliferation. While we agree that this is an interesting difference with our work, it represents a difference in experimental conditions employed and not, as implied, a difference in cellular responsiveness to PTHRP. Indeed, since Maoli and Fortino do not show a dose response experiment, it is unclear how their cells would respond to 125 nM PTHRP. On the same point, as stated in our paper, we did not detect any PTHRP-induced Ca^2+^ signal in parental MCF-7 cells treated with 125 nM PTHRP, but did not examine this at the higher ligand concentrations employed by Maioli and Fortino. We suggest, therefore, that there is no basis for their comment about the likely loss of this pathway in our cells. However, in the light of the data shown by Maioli and Fortino, it would clearly be interesting to conduct a more detailed dose–response analysis of the effects of PTHRP on mitogenesis in MCF-7 cells.

The third point raised by Maioli and Fortino alludes to our finding of an increased sensitivity to growth factors in cells overexpressing the PTH1R. We were, of course, aware of the vast literature on tyrosine kinase receptor (TKR) transactivation by G protein-coupled receptors and did not claim to be making a novel observation in that respect. We merely report the phenomenon, which we believe is interesting, but do not, at this stage, have substantive experimental evidence concerning its underlying mechanism. We can say however that our unpublished data indicate that the sensitivity observation is not due to TKR transactivation, and appears instead to involve a synergistic interaction of signalling pathways downstream of the two receptors.

The final point relates to the effects of forskolin on proliferation; our results indicate a positive effect of forskolin, whereas Maoli and Fortino report a negative effect. As stated in our paper, different concentrations of forkolin that lead to differences in both the peak level of cAMP induced as well as its temporal kinetics can affect cellular outcome. The promitogenic effects we observed were in reponse to 100 nM drug whereas Maioli and Fortino saw antiproliferative effects at 100 *μ*M forskolin. We suggested, on this same point, that such a phenomenon could also explain the apparent contradictory effects of expressing a constitutively active allele of G_s_ in MCF-7 cells (Chen *et al*., 1998).

In summary, although there appear to be a number of differences between aspects of our work and the preliminary findings of Maioli and Fortino, most of these appear to be based on differences in experimental approach. Therefore, it is not surprising that some divergent results have emerged. We would like to emphasise that the aim of our work was, in essence, to determine the effects of increased levels of PTH1R expression in breast cancer cells, aims which, though overlapping, are clearly divergent from those that could be addressed by the Maioli and Fortino study.

## References

[bib1] Chen J, Bander JA, Santore TA, Chen Y, Ram PT, Smit MJ, Iyengar R (1998) Expression of Q227L-galphas in MCF-7 human breast cancer cells inhibits tumorigenesis. Proc Natl Acad Sci USA 95: 2648–2652 9482941 10.1073/pnas.95.5.2648 PMC19449

[bib2] Falzon M, Du P (2000) Enhanced growth of MCF-7 breast cancer cells overexpressing parathyroid hormone-related peptide. Endocrinology 141: 1882–1892 10803599 10.1210/endo.141.5.7470

